# Rapid Detection of Hepatitis B Virus in Blood Samples Using a Combination of Polymerase Spiral Reaction With Nanoparticles Lateral-Flow Biosensor

**DOI:** 10.3389/fmolb.2020.578892

**Published:** 2021-01-07

**Authors:** Lin Lin, Jinshuai Guo, Haiyang Liu, Xiaofeng Jiang

**Affiliations:** General Surgery, The Fourth Affiliated Hospital of China Medical University, Shenyang, China

**Keywords:** hepatitis B virus, lateral flow biosensor, polymerase spiral reaction, rapid detection, clinical sample

## Abstract

A rapid, highly sensitive, and robust diagnostic technique for point-of-care (PoC) testing can be developed using the combination of the nanoparticle-based lateral flow biosensors (LFB) and isothermal nucleic acid amplification technology. Here, we developed a polymerase spiral reaction (PSR) containing FITC-labeled DNA probes coupled with the nanoparticle-based LFB assay (PSR-LFB) to detect the amplified products to detect HBV visually. Under the optimized conditions, the PSR assay involved incubation of the reaction mixture for 20 min at 63°C, followed by visual detection of positive amplicons using LFB, which would generate a red test line based on the biotin/streptavidin interaction and immunoreactions, within 5 min. A cross-reactivity test revealed that the developed PSR-LFB assay showed good specificity for HBV and could distinguish HBV from other pathogenic microorganisms. For the analytical sensitivity, the limit of detection (LoD) of PSR-LFB assay was recorded as 5.4 copies/mL of HBV genomic DNA, which was ten-times more sensitive than qPCR and loop-mediated isothermal amplification (LAMP). Additionally, all the HBV-positive (29/82) samples, identified using ELISA, were also successfully detected by the PSR-LFB assay. We found that the true positive rate of the PSR-LFB assay was higher than that of qPCR (100 vs. 89.66%, respectively), as well as the LAMP assay (100 vs. 96.55%, respectively). Furthermore, the integrated procedure could be completed in 60 min, including the processing of the blood samples (30 min), an isothermal reaction (20 min), and result visualization (5 min). Thus, this PSR-LFB assay could be a potentially useful technique for PoC diagnosis of HBV in resource-limited countries.

## Introduction

The combination of nanomaterials and biotechnology provides an excellent platform for the successful application of surface functionalization in several diagnostic techniques. Biosensors have been widely used for the identification of genetic mutations, environmental monitoring, and therapeutic diagnosis, especially for point-of-care (PoC) testing of communicable diseases (Riahi et al., 2011; Ahangar and Mehrgardi, 2017). Diagnosis at an early stage is necessary to prevent the transmission of viral diseases, which requires highly specific, sensitive, and cost-effective testing methods (Li et al., 2019). Due to their molecular properties, such as strong fluorescence and bio-conjugation ability, noble-metal nanoclusters (NCs) have been regarded as ideal materials for the preparation of nucleic acid-based biosensors (Li et al., 2015; Di Nardo et al., 2019). Among these, the gold nanoparticle-based lateral flow biosensors (AuNPs-LFB) are widely used for PoC testing to detect several pathogens due to their simple synthesis, relative stability, and visual analysis (Huang et al., 2016; Tomas et al., 2019). However, its limitations include non-specific binding of the analyte to the antibodies attached to the surface of AuNPs, along with the binding of these antibodies to other impurities, which results in a hypodynamic, when detecting analytes at low concentrations (Chen and Yang, 2015; Kang et al., 2019). Thus, a reliable, rapid, and sensitive amplification step is essential to improve the sensitivity of the reaction.

Hepatitis B virus (HBV), a circular, partially double-stranded DNA virus (approximately 3.2 kb) with eight different serotypes (A-H), is one of the leading cause of liver cirrhosis, hepatitis, and liver cancer (Shen et al., 2019). The WHO lists HBV as a global health issue with an estimated 2 billion people with active or hidden serological markers leading to approximately 700,000 deaths annually (Liu and Yao, 2015). This viral disease is transmitted through blood and body fluids and has a high rate of morbidity and mortality in infants and young children if untreated, especially in Asian and African countries. HBV attacks the human immune system, and thus, due to the scarcity of HBV-specific antiviral drugs, early screening is essential to control the spread of HBV (Franco et al., 2012). Currently, the HBV diagnosis is done using immunoassays [based on the interaction between HBV surface antigen (HBsAg) biomarkers and a soluble HBV antigen (HBeAg) using ELISA] and gene analysis (quantifies viral load, genotyping, and resistance mutations using real-time PCR) (Mao et al., 2016). Unfortunately, these methods are laborious and time-consuming and need sophisticated and expensive equipments, limiting their use in resource-limited settings.

Several isothermal nucleic amplification technologies (INATs), such as multiple cross displacement amplification (MCDA), loop-mediated isothermal amplification (LAMP), isothermal multiple-self-matching-initiated amplification (IMSA), and cross-priming amplification (CPA) have been successfully developed to detect DNA sequences in real-life samples in labs with basic facilities (Ding et al., 2015; Kuta et al., 2015; Liu et al., 2019b; Zhao et al., 2019). All these methods improve the specificity of the assay by employing a special primer set, which recognizes minimum six independent regions of the target gene, using *Bacillus stearothermophylus* (Bst) DNA polymerases to conduct *in situ* auto-cycling strand displacement DNA synthesis at 60–65°C using a simple heating device (Liu et al., 2019a). Nowadays, the INATs are being used for the detection of various pathogenic microbial infections like HBV (Ding et al., 2015), African Swine Fever Virus (Fraczyk et al., 2016), human papillomaviruses (HPV) (Livingstone et al., 2016), etc. Recently, another rapid, specific, and sensitive method i.e., isothermal polymerase spiral reaction (PSR), was developed (Liu et al., 2015). Unlike PCR and other INATs methods, which require DNA pre-denaturation at high temperature, special equipments, or complex primer combinations, PSR uses two specific primers with unrelated exogenous sequences and are independent of complex design to achieve large-scale amplification of the target genes in a short period. Amplification products can be identified and analyzed either by a real-time turbidity detector or visually by adding a fluorescent, double-stranded DNA binding dye (Dong et al., 2015; Xu et al., 2019).

Here, we developed a new simple, rapid, and ultrasensitive technique for the colorimetric detection of the HBV in human serum plasma using the PSR assay combined with the novel lateral flow biosensor (PSR-LFB). The PSR final products were dual labeled with FITC labeled probe and biotins, and were immobilized on the surface of colloidal gold nanoparticles to construct a DNA hybrid biosensor, which could be tested through the sandwich assay, followed by visual detection of the results. Thus, this detection tool could be used for the analysis of DNA-based disease, particularly in the PoC testing of blood samples.

## Materials and Methods

### Materials

Biotinylated bovine serum albumin (biotin-BSA), rabbit anti-fluorescein isothiocyanate (anti-FITC) monoclonal antibody (McAb), and streptavidin-immobilized 30 nm gold nanoparticles (SA-GNPs) were procured from Abcam Co. Ltd., China. Bst DNA Large Fragment Polymerase with 10 × thermopol buffer was obtained from New England Biolabs, United States. MgCl_2_, dNTPs (containing biotin-dUTP), and betaine were obtained from Shanghai Qifa experimental Reagent Co., Ltd., China). Hepatitis B virus Antigen (HBsAg) ELISA kit was procured from AmyJet Scientific Co., Ltd., China. SYBR Green I and PureLink Viral RNA/DNA kits were purchased from the Invitrogen, United States. The nitrocellulose (NC) membrane, backing card, absorbent pad, conjugate pad, and sample pad were acquired from Jieyi Biotechnology Co., Ltd., China. The gel image was captured using a Bio-Rad iQ5 system (Bio-Rad, CA). Nano-Photometer was bought from Bio-DL co., Ltd., China. A real-time turbidimeter was procured from Eiken Chemical Co., Ltd., Japan.

### Clinical Specimens, Standards, and DNA Preparation

We collected 82 specimens from the plasma of patients with suspected HBV who were admitted to the Fourth Affiliated Hospital of China Medical University between June 2017 and March 2019 to develop this assay. All the suspected HBV infected donors (42 male; 40 female) were aged between 28 and 46 years. As controls, ten archived healthy volunteers were selected from hospital employee list. The study procedure complied with the World Medical Association Declaration of Helsinki-Ethical Principles for Medical Research Involving Human Subjects and was approved by the Ethics Committee of the China Medical University (No: CMU62073024). The participants were informed regarding the objectives and benefits of the study following which a written informed consent form was obtained from each participant. Additionally, a conserved fragment of the S gene (approximately 680 bp) in HBV-PH4 was synthesized and cloned into the pAR2 expression vector as the positive control to determine the specificity and sensitivity of PSR-LFB. Other published reference strains, including hepatitis C virus (HCV), HPV, norovirus, etc., were purchased from BeNa Culture Collection, China (Table 1).

**TABLE 1:** Strains used in this study.

Species	Strain	PSR-LFB	LAMP	qPCR
Hepatitis B virus	HBV- PH4	+	+	+
Hepatitis B virus	HBV-06-1B	+	+	+
Hepatitis B virus	HBV-27-2A	+	+	+
Hepatitis A virus	BNCC310606	−	−	−
Hepatitis C virus	HM674631	−	−	−
Hepatitis C virus	HEBEI L02836	−	−	−
Hepatitis C virus	HK10U10197	−	−	−
Hepatitis C virus	HB-10592	−	−	−
Hepatitis C virus	HB-8064	−	−	−
Human papilloma virus	ATCC 40379	−	−	−
Norovirus	BNCC300545	−	−	−
*Mycobacterium tuberculosis*	ATCC 25177	−	−	−
*Staphylococcus aureus*	ATCC 25923	−	−	−
*Klebsiella pneumoniae*	ATCC 70603	−	−	−

**+, positive result; −, negative result.**

PureLink Viral RNA/DNA kits were used to isolate the genomic DNA from the viral extracts to prepare the DNA templates following the manufacturer’s protocol. Briefly, serum (25 μL) was diluted with nuclease-free water (50 μL), vortexed, and sequentially heated in a heating device for 5 min at 95°C, 5 min at 100°C, followed by centrifugation at 10,000 rpm for 3 min. The supernatant was retained, and the DNA concentration were measured using a NanoDrop spectrophotometer, aliquoted, and stored at −80°C until further use.

### Synthesis of Nanoparticle-Based Lateral Flow Biosensor (LFB)

**FIGURE 1:**
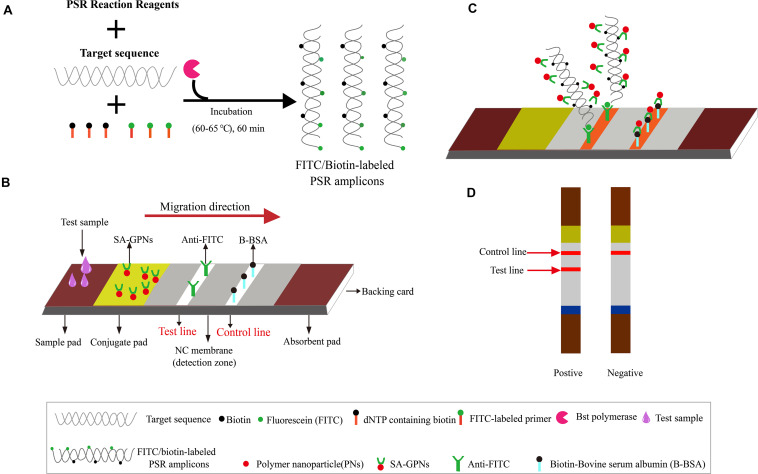
The outline of PSR combined with nanoparticle-based LFB assay (PSR-LFB). **(A)** The brief overview of PSR amplification process. **(B)** Schematic diagram of the assembly of nanoparticle-based LFB device. **(C)** Schematic of the LFB for visualizing PSR amplicons. **(D)** Analysis of the PSR-LFB assay: appearance of dual red bands in the visual region of LFB confirmed positive result, while one red band in the CL indicated a negative result.

The nanoparticle-based LFB dipstick was constructed following a previously described method using a conjugate pad, an immersion pad, an absorbent pad, and an NC membrane. The commercial SA-GNPs were soaked in 0.01 M phosphate buffer (pH 7.4), followed by dropwise addition to the conjugate pad at 100 μL/cm^2^ for deposition. After drying for 2 h at 37°C, the biotin-BSA and anti-FITC McAb, 1 mg/mL each, were immobilized on the NC membrane at 2 μL/cm^2^ as the control line (CL) and the test line (TL) (Wang et al., 2017a) at a separation distance of 3–5 mm, respectively. Then, the biosensor was assembled by sequentially gluing the sample pad, the conjugated pad, the NC membrane, and the absorption pad onto a plastic adhesive black blacking card with 1–2 mm overlapping, followed by slicing into 3 mm wide sections (Figure 1). All biosensors were stored at 4°C in plastic cassettes with a desiccant until use.

### Design of Primers for PSR Assay

**FIGURE 2:**
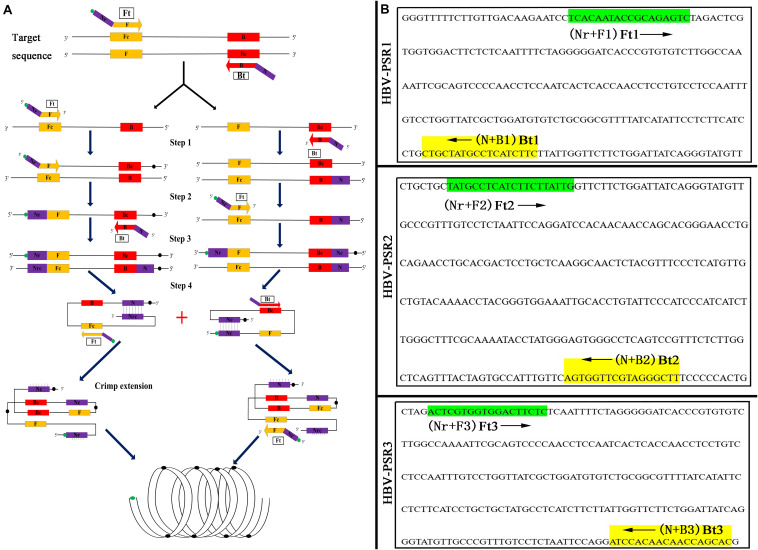
Schematic and primers location of PSR assay. **(A)** Schematic diagram of the PSR with FITC label. **(B)** Binding location for three groups of PSR specific primers. The arrowed lines indicate the direction of extension of the primer.

According to National Center for Biotechnology Information (NCBI) Blast analysis of the S protein (S) gene sequence in HBV (GenBank accession number AB116094.1), three sets of primers containing unrelated exogenous complementary sequences (Nr and N sequences) and an FITC-labeled probe were designed using Primer Premier 5.0 based on the highly conserved regions. Figure 2 and Table 2 provide a list of the primer sequences, locations, and concise schematic diagram of the PSR primers. Furthermore, a set of LAMP primers (Nyan et al., 2014) and a pair of qPCR primers (Kania et al., 2014) were added to further assess the specificity and sensitivity of this PSR method. All specific primers were synthesized and purified at HPLC grade purity by Sangon Biotech Ltd., China.

**TABLE 2:** Primers of PSR, LAMP, and qRT-PCR assays.

Name	Primer ID	Primers Sequence (5′–3′)	Nucleotide Length
HBV-PSR1	Ft1	gtcaaagcgatcccgccttac-TCACAATACCGCAGAGTC	39
	Bt1	cattccgccctagcgaaactg-GAAGATGAGGCATAGCAG	39
HBV-PSR2	Ft2	gtcaaagcgatcccgccttac-TATGCCTCATCTTCTTATTG	41
	Bt2	cattccgccctagcgaaactg-AAGCCCTACGAACCACT	38
HBV-PSR3	Ft3	gtcaaagcgatcccgccttac-ACTCGTGGTGGACTTCTC	39
	Bt3	cattccgccctagcgaaactg-GTGCTGGTTGTTGTGGAT	39
LAMP	HBU-FIP	GTTGGGGACTGCGAATTTTGGC-TTTT-TAGACTCGTGGTGGACTTCT	46
	HBU-RIP	TCACTAACCAACCTCCTGTCCT-TTTT-AAAACGCCGCAGACACAT	44
	HBU-F3	TCCTCACAATACCGCAGAGT	20
	HBU-B3	GCAGCAGGATGAAGAGGAAT	20
	HBU-LF	GGTGATCCCCCTAGAAAATTGAG	23
	HBU-LR	AATTTGTCCTGGTTATCGCTGG	22
qPCR	HBV-TAQ1	GTGTCTGCGGCGTTTTATCA	20
	HBV-TAQ2	GACAAACGGGCAACATACCTT	21
	TaqMan probe	CCTCTTCATCCTGCTGCTATG CCTCATC	28

### Optimization of the PSR Assay

The PSR assay was performed under standard amplification conditions at 60°C for 60 min using 25 μL reaction mixture containing: 4.0 μL each of amplification primers (10 mM), 2.5 μL of 10x ThermolPol reaction buffer, 2 μL of MgCl_2_ (3 mM), 2.5 μL of dNTPs (10 mM), 1 μL *Bst* DNA polymerase large fragment (8 U), 2.0 μL of betaine (1.0 M), 2.0 μL of an appropriate concentration of standard DNA (approximately 30 ng), and 5 μL of sterilized deionized water. Subsequently, the incubation temperatures (61–71°C) and the following reaction system components: *Bst*-DNA polymerase (6–12 U), MgCl_2_ (1.0–3.0 mM), betaine (0.6–1.2 M), and dNTPs (1.0–3.0 mM), were optimized under fixed conditions for 60 min, followed by incubation for 5 min at 85°C to terminate the PSR reactions. Finally, the amplicons were detected using any of these four different methods: turbidity monitoring using a real-time turbidimeter, separation using 2% agarose gel electrophoresis (AGE), direct visual detection using SYBR Green I (SGI, 2,000x) dyestuff, or LFB. For the LFB assay, PSR amplicon products (5 μL) mixed with 10 mM PBS (95 μL; pH 7.4 with 1% Tween 20) were deposited on the sample pad and the visual detection was done within 15 min based on the formation of red TLs on the NC membrane (the negative test resulted in the appearance of CLs and the positive test showed the CLs and TLs. If the CL was absent, the test was considered invalid). All the amplification reactions were performed in triplicates to ensure reproducibility.

For the LAMP assay, the reaction was performed at 60°C for 60 min, following the method of Nyan et al. (2014), using 25 μL reaction mixture containing the inner primers (HBU-FIP/HBU-BIP; 1.2 μmol/L each), the Loop primers (HBU-LF/HBU-LR; 0.8 μM each), and the outer primers (HBU-F3/HBU-B3; 0.4 μmol/L each); dNTPs (2.0 mM), MgSO4 (1 mM), 10 × Bst polymerase buffer (2.5 μL), betaine (0.8 M), Bst DNA large fragment polymerase (8 U), and target DNA template (2 μL). The real-time PCR was conducted in the Bio-Rad iQ5 system, following the manufacturer’s guidelines. The thermal cycling parameters were as follows: initial denaturation at 95°C for 10 min, followed by 40 cycles at 95°C for 15 s and 60°C for 1 min. All the amplification reactions were performed in triplicates.

### The Analytical Sensitivity of the PSR-LFB Assay

Ten-fold serial dilutions of the standard template HBV-HP4 were synthesized to evaluate the LoD of the PSR-LFB assay, to compare its sensitivity with other molecular techniques, including LAMP and qPCR assays. The analytical sensitivity of PSR-LFB was assessed by 2% AGE, SGI fluorescence dyestuff, and LFB assay as described previously, while the LAMP assay was confirmed by 2% AGE and SGI analysis. These detection methods were performed in triplicates to verify the reproducibility. The LoD of each method was determined based on the last dilution value before the first negative result appeared in three replicates. Also, we optimized the time parameter of the PSR-LFB assay simultaneously. Each dilution was tested in triplicates.

### The Analytical Specificity of the PSR-LFB Assay

Three strains of HBV (HBV-PH4 and 2 clinical isolates) and 11 non-HBV standard strains (HCV, HPV, norovirus, *Mycobacterium tuberculosis*, *Klebsiella pneumoniae*) were tested by the PSR, LAMP, and qPCR assay under previously described conditions. The PSR amplicons were detected using 2.0% AGE and LFB assay, while the LAMP products were analyzed using 2.0% AGE and SGI (2,000x). Each experiment was performed in triplicates.

### HBV Detection From Clinical Specimens

We collected 82 blood specimens with suspected HBV infection and ten healthy blood samples from Liaoning province in China between 2017 and 2019. All the HBV-negative specimens were tested for HBsAg using an ELISA kit following the manufacturer’s guidelines and 29 HBsAg-positive (17 with genotype B; 9 with C, and 3 with D, determined using the AI-HBV-Cap Enrichment Kit^®^ on the OpenGene DNA Sequencing System) serum samples were identified. Then, DNAs were extracted using commercial kits as previously described and the 2 μL of each supernatant was used for PSR-LFB, LAMP, and qPCR assays following the optimized conditions following the previously described methods. Each experiment was performed in triplicates and the consistency rate of the qPCR, PSR-LFB, or LAMP assay was compared with the results of ELISA.

## Results

### Optimization of the PSR Assay

**FIGURE 3:**
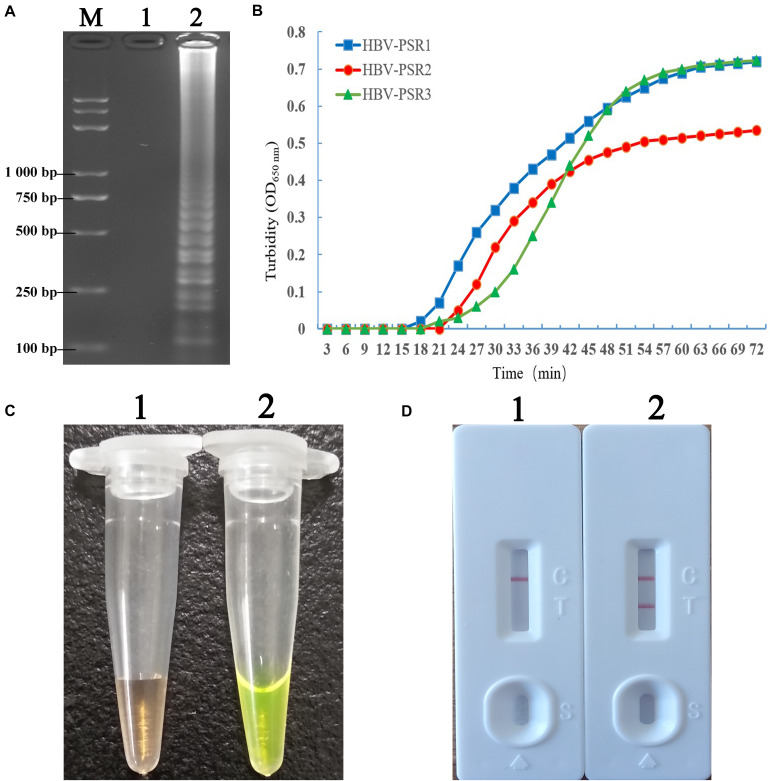
Detection and confirmation of PSR amplicons with the appropriate primer combination. **(A)** 2% AGE of the PSR amplified products. **(B)** Observation of real time turbidity for the following primer combinations: HBV-PSR1, HBV-PSR2, and HBV-PSR3. **(C)** Observation of the PSR-amplified products mixed with SGI under natural light. **(D)** Analysis of the PSR products by the LFB assay. M: DNA maker DL8000. 1, negative control; 2, Amplification of HBV-PH4. LFB, lateral flow biosensor; SGI, SYBR Green I dye.

Figure 2A illustrates the development and mechanism of the PSR assay (Xu et al., 2019). Here, we designed three sets of HBV-PSR specific primers for the highly conserved regions of the S gene sequences, and the amplification was done following the previously described method. For the AGE analyses using ethidium bromide (EB), a typical ladder like pattern was observed for HBV positive amplifications, while no band was observed in HBV negative amplifications (Figure 3). Simultaneously, the results of real-time monitoring by a turbidimeter, SGI dyestuff, and LFB assay showed that the primer combination of HBV-PSR1 had the best amplification. Additionally, no difference was observed in the amplification of PSR primers using modified and unmodified probes, indicating that biotin and FITC did not interfere with the reaction system and could be used for further experimental analysis.

**FIGURE 4:**
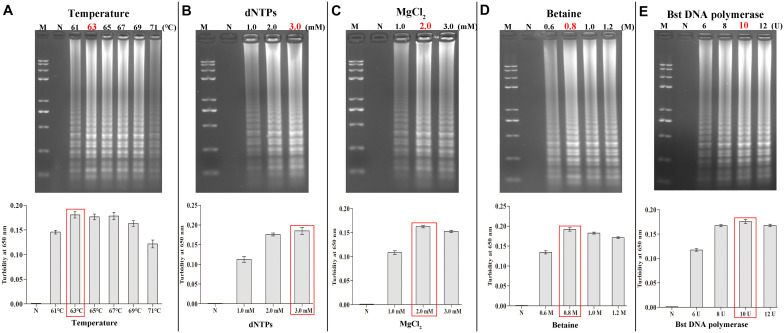
Optimal amplification conditions for HBV-PSR assay. The optimal PSR reaction conditions to detect HBV-PH4 were monitored by 2% AGE and real-time turbidity. **(A)** Incubation temperature optimization. **(B–E)** Concentration optimization for dNTPs, MgCl_2_, betaine and Bst DNA polymerase, respectively. M: DNA maker DL8000; N: negative control.

Next, the incubation temperature and the system components were consecutively optimized using the 2% AGE and a real-time turbidimeter. Using HBV DNA as the template (100 ng/μL), we found 63°C to be the optimal temperature during amplification (Figure 4A). At a fixed incubation temperature of 63°C for 60 min, the following optimized reaction conditions were obtained: 3.0 mM dNTPs, 0.8 M betaine, 2.0 mM MgCl_2_, and 10 U/tube of Bst DNA polymerase (Figures 4B–E).

### Analytical Sensitivity of PSR-LFB Assay

**FIGURE 5:**
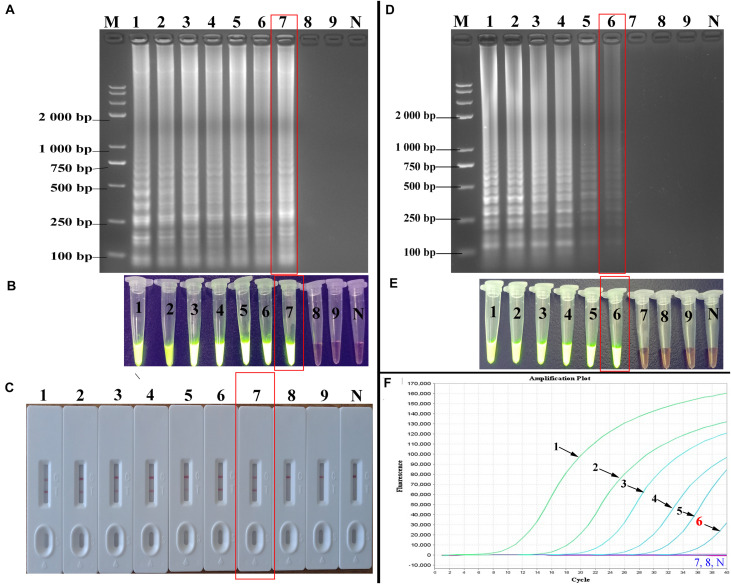
Sensitivity of the PSR-LFB assay for HBV detection. **(A–C)** Analysis the PSR amplicons by AGE, SGI, and LFB assay, respectively. **(D,E)** Amplification results of LAMP assay by AGE and SGI method. **(F)** Detection of HBV by qPCR. M: DNA maker DL8000. Lanes/tubes/biosensors/fluorescence signals 1–9: 5.4 × 10^6^, 5.4 × 10^5^, 5.4 × 10^4^ 5.4 × 10^3^, 5.4 × 10^2^, 5.4 × 10^1^, 5.4 × 10^0^, 5.4 × 10^–1^, 5.4 × 10^–2^ copies/mL, respectively. N: negative control.

At 10-fold serial dilution (from 5.4 × 10^6^ to 5.4 × 10^–2^ copies/mL) of HBV genomic DNA was done to compare the sensitivity of the PSR-LFB assay with the LAMP and qPCR techniques (Figure 5). First, the PSR products were analyzed by 2% AGE and stained with EB. The sensitivity of PSR assay for HBV detection was found to be 5.4 copies/mL, which is equivalent to approximately 1.0 IU/reaction (Figure 5A). Using the SGI dyestuff and biosensor, the positive products of HBV-PSR assay were directly observed as bright green fluorescence under the UV light (Figure 5B) and exhibited two clear red lines (Figure 5C), while the negative sample remained orange and generated a single red line. Concurrently, the different dilutions of HBV genomic DNA mentioned above were detected by LAMP and qPCR assay, and the detection limits were found to be 5.4 × 10^1^ copies/mL, (Figures 5D,E). These results showed that the detection sensitivity of the PSR-LFB assay was ten times higher than that of the LAMP and qPCR detection techniques.

### The Time Optimization of the PSR-LFB Assay

**FIGURE 6:**
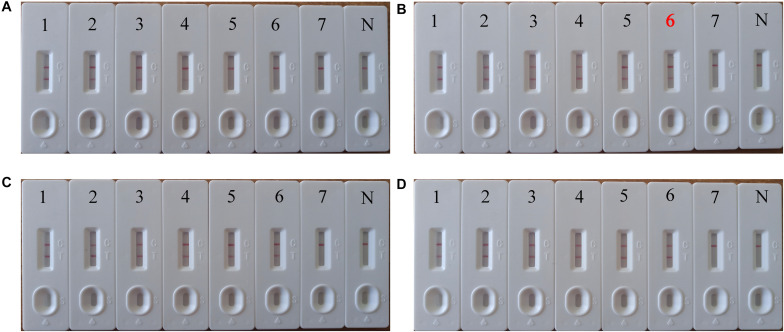
The optimal incubation time required for PSR-LFB assay. **(A–D)** Four distinct incubation times (10, 20, 30, and 40 min) were compared at 63°C. Biosensor 1–7 correspond to the following DNA levels: 5.4 × 10^5^, 5.4 × 10^4^, 5.4 × 10^3^, 5.4 × 10^2^, 5.4 × 10^1^, 5.4 × 10^0^, 5.4 × 10^–1^ copies/mL. N: negative control. The best sensitivity at 20 min has been marked with red **(B)**.

We systematically analyzed the optimal time required to perform the PSR-LFB assay during the PSR amplification stage to achieve rapid detection. Various reaction times (10–40 min at 10 min intervals) were employed at 63°C following the standard PSR reaction systems (Figure 6). The LoD (5.4 copies/mL of HBV templates) showed dual red bands for a reaction time of 20 min at 63°C; thus these conditions were regarded as the optimal conditions for the PSR-LFB assay (Figure 6B). Thus, the whole process including DNA extraction of blood samples (30 min), isothermal reaction (20 min) and visualization of results (10 min) could be accomplished within 60 min.

### Analytical Specificity of PSR-LFB Assay

**FIGURE 7:**
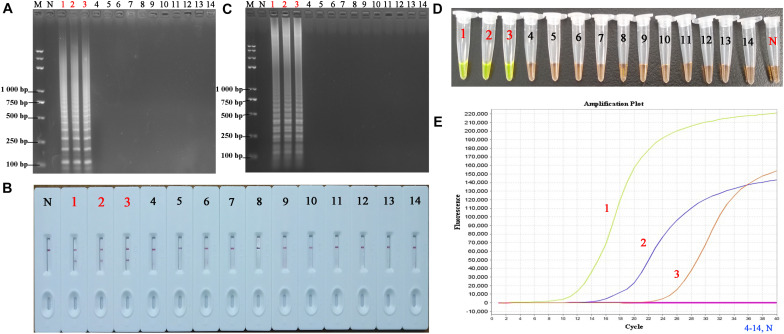
The specificity of PSR-LFB assay compared with LAMP and qPCR assay. **(A,B)** The PSR amplicons were amplified using distinct genomic DNA templates and analyzed by AGE and LFB. **(C,D)** Analysis of the products from the LAMP assay using AGE and SGI under UV light, respectively. **(E)** Amplification results of qPCR. M, DNA maker DL8000; N, negative control. 1–3, HBV- PH4, HBV-06-1B, HBV-27-2A; 4, Hepatitis A virus; 5–9, Hepatitis C virus; 10, Human papilloma virus; 11, norovirus; 12, *Mycobacterium tuberculosis;* 13, *Staphylococcus aureus*; 14, *Klebsiella pneumonia.*

The specificity of the PSR-LFB assay was assessed by testing the genomic DNA isolated from three HBV strains and 11 non-HBV strains, respectively. All products derived from the genomic DNA of HBV displayed dual red bands on the LFB, while only one red band for each sample for the non-HBV and blank control (Figure 7). We observed no cross-contamination, false-positive, or false-negative results while performing this PSR-LFB assay. Thus, the PSR-LFB assay showed 100% specificity for the detection of HBV, consistent with the results of LAMP (obtained from electrophoresis analysis and SGI method) and the qPCR assay.

### Clinical Application of PSR-LFB

**FIGURE 8:**
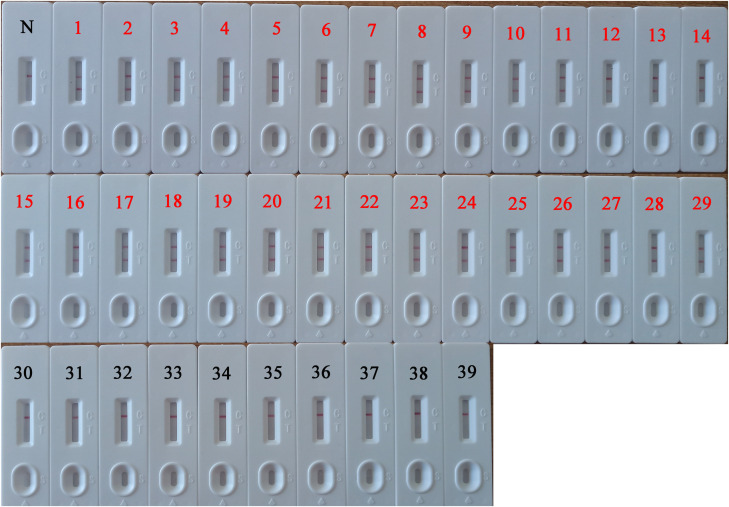
PSR-LFB assay for the detection of HBV in blood specimens. 1–29: The 29-positive results of PSR-LFB assay in 82 clinical blood samples with suspected HBV infection. 30–39: Detection results of 10 healthy blood samples by PSR-LFB assay. N, negative control.

The DNA isolated from 82 HBV-suspected clinical blood specimens and 10 healthy blood specimens were analyzed by LAMP, qPCR, and PSR-LFB assay, respectively to evaluate the feasibility of PSR-LFB assay as a reliable detection tool for the HBV. Using the ELISA method, 29 (35.37%) of the 82 blood specimens were found to be HBV positive, and the diagnostic accuracy obtained was in complete agreement with the PSR-LFB assay (Figure 8). On the contrary, out of the 29 positive specimens, the LAMP and qPCR assay identified 26 (89.66%) and 28 (96.55%) specimens as HBV positive, respectively (Table 3). These results confirmed that this PSR-LFB assay could be used for PoC diagnostics compared with the LAMP and qPCR method.

**TABLE 3:** Detection results of 82 clinical blood samples of suspected HBV amplified by four different methods.

Age (years)	Sex		ELISA (*n* = %)		PSR-LFB (*n* = %)		LAMP (*n* = %)		qPCR (*n* = %)	
	Male	Female	Positive	Negative	Positive	Negative	Positive	Negative	Positive	Negative
18∼27	21	19	17 (20.73)	23 (28.05)	17 (20.73)	23 (28.05)	16 (19.51)	24 (29.27)	17 (20.73)	23 (28.05)
28∼37	15	12	9 (10.98)	18 (21.95)	9 (10.98)	18 (21.95)	7 (8.55)	20 (24.39)	8 (9.76)	19 (23.17)
38∼47	6	9	3 (3.66)	12 (14.63)	3 (3.66)	12 (14.63)	3 (3.66)	12 (14.63)	3 (3.66)	12 (14.63)
Total	42	40	29 (35.37)	53 (64.63)	29 (35.37)	53 (64.63)	26 (31.71)	56 (68.29)	28 (34.15)	54 (65.85)

## Discussion

The high prevalence and mortality of HBV infection remains a public health issue that demands attention especially in the underprivileged communities and regions of the world (Rybicka et al., 2016). However, ELISA-based diagnosis of HBV presents time and cost-related constraints in resource-limited units; thus, limiting its early detection (Chen et al., 2019; Shen et al., 2019). Although INATs, including real-time PCR, nested-PCR, and LAMP are commonly used to detect HBV in blood; however, tedious operation, expensive equipment, and cross contamination limits their application (Akram et al., 2018). Thus, a simple, reliable, and rapid screening and diagnostic method needs to be developed that is not only applicable to the PoC diagnosis but also fulfils the ASSURED criteria (affordable, sensitive, specific, user-friendly, rapid/robust, equipment-free, and deliverable) in regions with high prevalence of HBV. Nowadays, the sensitivity, cost effectiveness, and distinct physical and chemical characteristics of LFB enable its use as a superior analysis strategy for PoC testing (Petrakova et al., 2019). Therefore, we developed an assay to detect HBV S gene using LFB conjugated with PSR (PSR-LFB), where a pair of primers and probes were employed to detect the target sequence and form the corresponding biocompatible amplicons, which act as a new fiducial mark identified by a standardized biosensor. The specific biosensor could detect the DNA templates extracted from HBV-positive strains, but not the non-HBV isolates and blank control. Compared with the traditional strip or biosensor analysis, the PSR-LFB assay does not require specific antibody of the pathogenic microorganism, thus, improving the specificity and sensitivity of the whole process to a certain extent.

Though several isothermal amplification methodologies have greatly facilitated clinical screening and PoC diagnosis, such as LAMP, IMSA, MCDA, etc., they usually require multiple specific primers within a limited number of gene segments (300 bp) for accurate detection. However, designing specific primers or the frequent mismatch between primers limits their application. Thus, this study used a recently established PSR method to detect HBV by targeting the S gene. This assay provided a simplified format for nucleic acid detection, with a design principle similar to the PCR method, but used the Bst polymerase to amplify target sequence under isothermal condition to enhance the efficiency of DNA amplification. The reaction system conditions of PSR were optimized at 63°C with the concentration of Bst-DNA polymerase as 10 U, dNTP as 3.0 mM, betaine as 0.8 M, and MgCl_2_ as 2.0 mM, which were consistent with the traditional conditions. However, in terms of monitoring techniques, AGE, turbidimetric analysis, and intercalation dyes for real-time detection have achieved good effects, but additional gel electrophoresis steps, special fluorescent reagents, and lack of recognition ability for specific amplification, and non-specific amplification limits their use in several fields. Thus, we used probes, including FITC and biotin in this PSR-LFB assay, based on the conventional PSR method. During the amplification stage, we constructed the dual-labeled detectable amplicon (biotin-dsDNA-FITC) using an FITC-labeled primer and biotin from the reaction system, which could be captured by the anti-FITC McAb fixed on the biosensor TL, while the other end of the biotin-labeled amplicon attached to the streptavidin-bound gold nanoparticles to form a positive band. Simultaneously, the excess streptavidin-conjugated gold nanoparticles were captured with biotinylated BSA to form the CL to validate the working conditions of the LFB. This PSR-LFB assay could detect amplification products within 5 min and was found to be more reliable than gel electrophoresis, turbidimetry, and visual detection.

Next, the specificity of PSR-LFB was confirmed using genomic DNAs of different pathogenic microorganisms. The results demonstrated that the PSR-LFB assay could identify all HBV-positive isolates without any cross-reaction and all non-HBV strains displayed a negative outcome, consistent with the LAMP assay. All these data indicated that the proposed assay showed high screening ability for the target DNA and could be used as a highly specific tool for HBV identification. Apart from specificity, the determination of detection limit is also very important for the PSR-LFB assay. Based on the results of the sensitivity analysis, we found that PSR-LFB could detect 5.4 copies/mL of HBV DNA, which was further confirmed by gel electrophoresis, SGI visual detection, higher than the LAMP and qPCR assay. Next, the PSR-LFB assay was performed on 82 HBV-suspected blood samples and compared with LAMP, qPCR, and ELISA assays. We found that the PSR-LFB and ELISA methods showed a higher detection sensitivity for HBV in the blood samples compared with the LAMP and qPCR assay (Table 3). The lower detection by the LAMP and qPCR assay could be due to sequence mismatch between the designed primers and the current virus strain, which also indicated that HBV was slowly undergoing evolution and variation. This indicated that the surveillance of HBV should be based on continuous epidemiological surveys and timely primer updates in different countries and regions. Additionally, the cost of PSR-LFB assay was determined based on the cost of the reagents, consumables (DNA Extraction Reagent), and primers along with the depreciation of equipment and the technologist’s salary of $2.5/h in China. The costs were based on a run size of 20 samples in each assay format. The PSR-LFB assay was economically advantageous for the identification of HBV compared with the qPCR assay ($4.5 vs. $7.3 per reaction). Also, the entire PSR-LFB procedure, including DNA preparation (30 min), isothermal amplification (20 min), and LFB reading (5 min), was accomplished within 60 min, without requiring skilled technical personnel and labor costs. Thus, our study demonstrated that the PSR-LFB assay could be used for the rapid, effective, accurate, and cost-effective detection of HBV in blood samples.

One of the limitations of this method is the opening of the reaction tube to analyze the PSR amplicons using LFB. Due to the high sensitivity of the PSR assay, once the reaction tube has been opened, the high concentration of aerosol droplets will produce false positive results. Thus, aerosol pollution presents a limitation in the field of nucleic acid amplification. Currently, many methods, such as paraffin oil sealing and specially designed reaction tube, have been adopted to avoid the risk of aerosol pollution during visualization, with encouraging results. Additionally, it has been reported that adding Antarctic thermolabile uracil-DNA-glycosylase (UNG) to the reaction system may remove the carried-over contamination (Wang et al., 2017b). Future studies would involve development of procedures to eliminate the residual pollution. Furthermore, the high sensitivity of the selected Bst DNA polymerases toward blood components would reduce the influence of other interfering components in clinical samples, providing an additional advantage for blood screening. Thus, a simple, time-efficient and highly sensitive PSR-LFB methodology was developed combining the advantages of isothermal amplification and biological nanomaterials to provide a powerful tool for monitoring HBV in resource-limited areas.

## Data Availability Statement

All datasets presented in this study are included in the article/supplementary material.

## Ethics Statement

The studies involving human participants were reviewed and approved by the Ethics Committee of China Medical University. The patients/participants provided their written informed consent to participate in this study.

## Author Contributions

LL and XJ conceived of and designed the experiments. LL, JG, HL, and XJ performed the experiments. LL and HL analyzed the data. LL and JG wrote the manuscript. XJ edited the manuscript. All authors contributed to the article and approved the submitted version.

## Conflict of Interest

The authors declare that the research was conducted in the absence of any commercial or financial relationships that could be construed as a potential conflict of interest.
